# Undernutrition and associated factors among internally displaced lactating mothers in Sekota camps, northern Ethiopia: A cross-sectional study

**DOI:** 10.3389/fnut.2023.1108233

**Published:** 2023-02-14

**Authors:** Misganaw Asmamaw Mengstie, Mulugeta Dile Worke, Yalemshet Belay, Endeshaw Chekol Abebe, Tadesse Asmamaw Dejenie, Mohammed Abdu Seid, Muluken Teshome Azezew

**Affiliations:** ^1^Department of Biochemistry, College of Health Sciences, Debre Tabor University, Debre Tabor, Ethiopia; ^2^Department of Midwifery, College of Health Sciences, Debre Tabor University, Debre Tabor, Ethiopia; ^3^Department of Physiology, College of Health Sciences, Debre Tabor University, Debre Tabor, Ethiopia; ^4^Department of Biochemistry, College of Medicine and Health Sciences, University of Gondar, Gondar, Ethiopia

**Keywords:** lactating mothers, internally displaced people, Sekota, Ethiopia, undernutrition

## Abstract

**Background:**

Undernutrition is the term used to describe when a person consumes insufficient amounts of nutrients and energy to meet their needs for maintaining health. Despite substantial progress, undernutrition remains a serious public health concern in many low and middle-income nations, including Ethiopia. Women and children are, in reality, the most nutritionally vulnerable individuals, particularly in times of crisis. In Ethiopia, 27 percent of lactating women are thin or malnourished, and 38% of children are stunted. Although the issue of undernutrition may worsen in times of emergency, like war, there are limited studies available in Ethiopia that show the nutritional status of lactating mothers in humanitarian settings.

**Objectives:**

The main aim of this study was to determine the prevalence and investigate the factors associated with undernutrition among internally displaced lactating mothers in Sekota camps, in northern Ethiopia.

**Methods:**

A cross-sectional study through a simple random sampling technique was conducted among 420 randomly selected lactating mothers in Sekota Internally Displaced Persons (IDP) camps. Data were collected using a structured questionnaire and anthropometric measurements. Logistic regression analysis was employed to identify independent factors associated with maternal undernutrition.

**Results:**

Using a cut-off mid-upper arm circumference <23 cm, the prevalence of undernutrition among internally displaced lactating mothers was 54.8%. Large family size [adjusted odds ratio (AOR) = 4.35; 95% CI: 1.32, 10.22], short birth interval (AOR = 4.85; 95% CI: 1.24, 10.00), low maternal daily meal frequency (AOR = 2.54; 95% CI: 1.12, 5.75), and low dietary diversity score (AOR = 1.79; 95% CI: 1.03, 3.10) were all significantly associated with undernutrition.

**Conclusion:**

The prevalence of undernutrition among internally displaced lactating mothers is relatively high. Governments and other concerned organizations involved in providing care and support to Sekota IDP camps should increase their efforts to improve the nutritional status of lactating mothers

## Background

Ethiopia is Africa’s oldest independent country and it is the second largest in terms of the population (1). Ethiopia in the northern area has experienced internal conflict during the last 2 years between the government of Ethiopia and forces in its northern Tigray region (2). The conflict has had severe impacts on the civilian population. Many families have been forced to flee their homes (internally displaced) because of the armed conflict. Internally Displaced Persons (IDP) are people who have been forced to leave their homes due to war or other circumstances but do not meet the legal standards of refugees (3). Conflicts have a negative influence on food security by causing huge displacements, severe economic downturns, increased inflation and unemployment, and eroding funds for social protection and healthcare (4). Armed conflict is a significant contributor to the rising burden of malnutrition, resulting from decreased food availability, social disruption, higher food prices, and, eventually, starvation and/or disease. Internally displaced women and children are, in reality, the most vulnerable and conflict-affected individuals in the world (5). Generally, malnutrition among vulnerable and marginalized groups (including lactating women) because of armed conflict is a multifaceted problem that requires a multidisciplinary remedy (6).

Undernutrition is the term used to describe when a person consumes insufficient amounts of nutrients and energy to meet their needs for maintaining health (7). Despite substantial progress, malnutrition remains a serious public health concern in many low and middle-income nations, including Ethiopia, particularly in times of crisis (8). Due to their greater nutritional needs and the harmful consequences of inadequate nutrition on the health of both mothers and their children, lactating women and their children are among the most susceptible categories of the population during emergencies such as war (9). Undernutrition is a serious issue among lactating mothers in developing nations, particularly in Sub-Saharan Africa (10). In Ethiopia, both maternal and child malnutrition are common problems. Generally, 27 percent of Ethiopian women are thin or malnourished, and 38% of children are stunted (11). The prevalence of malnutrition among lactating women in Ethiopia varies by area, as does the anthropometric measurement technique. Some studies utilize mid-upper arm circumference (MUAC), whereas others employ body mass index (BMI). A community-based survey in Ethiopia’s Afar region revealed that 33.3% of mothers were malnourished based on MUAC (12). Using BMI, 21.2 and 17.4% of lactating mothers in the Angecha and Arba Minch districts of southern Ethiopia, respectively, were undernourished (13, 14). Malnutrition among lactating women was reported to be 21.8% in the Dega Damot district (15) and 21% in Dessie town (16) in northern Ethiopia. Another study in the Bale Zone of Oromia regional state found that 24% of lactating mothers living in humanitarian settings were malnourished (17). Although both BMI and MUAC can be used to determine undernutrition in adults, MUAC has long been used as a community-based assessment of undernutrition because it is easier to implement than BMI. Prior research also suggests that MUAC can be an effective indicator of female adult malnutrition, comparable to or even better than BMI (18, 19). Most published research uses the terms malnutrition and undernutrition interchangeably, while malnutrition is a broader term that includes both over-nutrition and undernutrition (20). However, undernutrition is the focus of this study. Although there have been a few studies to assess the nutritional status of lactating mothers in various regions of Ethiopia, screening, and management of malnutrition in humanitarian settings is limited, and there is no published evidence reporting maternal nutritional status in most conflict-affected areas in Ethiopia, particularly to the study setting. Furthermore, it is critical to identify the issue of undernutrition and its risk factors among people living in humanitarian settings, as this could help the government and other humanitarian organizations in designing their interventions. Hence, the objective of this study was to determine the prevalence and investigate the risk factors of undernutrition among internally displaced lactating mothers in Sekota camps, in northern Ethiopia.

## Materials and methods

### Study design, setting, and period

A cross-sectional study design was employed in IDP camps found in Sekota town. Sekota is the capital city of the Waghimra zone, Amhara region in northern Ethiopia. It is 436 and 876 kilometers away from Bahir Dar (the capital city of the Amhara region) and Addis Ababa (the capital city of Ethiopia), respectively. Sekota town is bounded by Gazbibla on the south, Zikuala on the west, Abergele on the north, and the east by the Tigray region. There are currently three temporary IDP camps in the town. These are Weleh, Mindikri, and Tirki. The town currently encompasses more than 65, 000 IDPs in all camps. Of these IDPs, around 3,000 residents are known to be lactating mothers, 4,200 are pregnant and 16,000 children less than 5 years of age collectively live in Sekota camps. The study was conducted from June 3 to July 10, 2022.

### Sample size determination

The minimum sample size required for the study was determined by using Epi info version 7.2 StatCalc software with the following assumptions. 95% CI, 5% margin of error, the number of lactating mothers found in Sekota IDP camps, which was 3,000, a 24% prevalence of undernutrition among lactating women in humanitarian settings from a previous study conducted in Ethiopia (17), and a design effect of 1.5. The calculated sample size was 384. Taking 10% of the possible non-response rate into account, the final sample size for the study is 422.

### Sampling procedures

**FIGURE 1:**
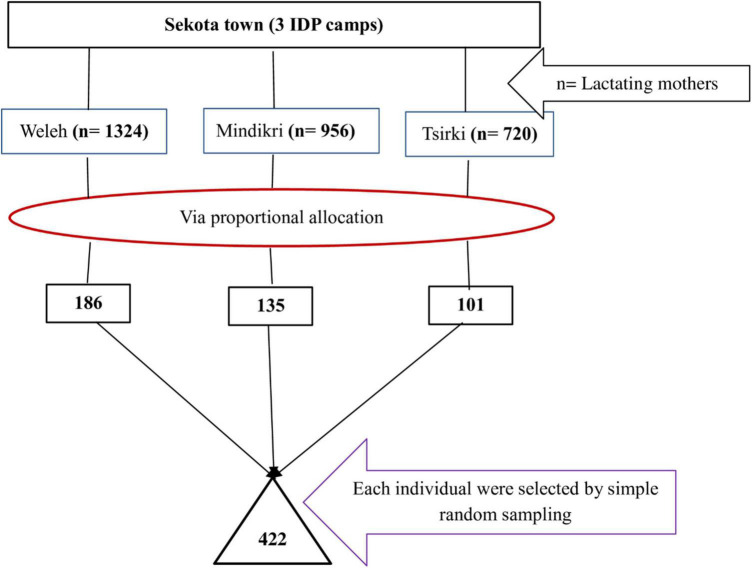
Schematic presentation of the sampling procedure for the study conducted among lactating mothers in Sekota camps, Ethiopia, 2022.

As a first step, a camp registration list of lactating mothers was identified using information from a rural health center. The total sample was then proportionately allocated to each camp. Then, using a computer-generated method at the household level, simple random sampling techniques were used to select study participants (Figure 1). When an eligible participant was not identified at their assigned campsite, the interviewers returned to the site at a later time during the data-collecting period.

### Eligibility criteria

The source population was all internally displaced lactating mothers living in all Sekota camps. The study population was randomly selected from internally displaced lactating mothers during the specified study period. Lactating mothers who visited the IDP camp during the study period were excluded from the study.

### Data collection tools and procedure

Data were collected using an interviewer-administered structured questionnaire and anthropometric measurement. The questionnaire has three sections including sociodemographic variables, maternal reproductive and healthcare-related questions, and dietary diversity-related questions. The individual dietary diversity of lactating mothers was evaluated using the qualitative recall of the mothers’ food consumption over the previous 24-h period. The tool includes 10 food groups (starchy staples, legumes and nuts, dairy, organ meat, any egg, meat poultry and fish, dark green leafy vegetables, other vitamin-A rich fruits and vegetables, other vegetables, and other fruits). It was then determined using a simple count and sum of the number of food groups consumed by each respondent during the prior 24-h recall period.

The MUAC of each lactating mother was measured using non-elastic, non-stretchable adult-size MUAC tapes at the midpoint between the points of the shoulder and elbow of the left upper arm. Since the development of the triceps and biceps muscles may be asymmetrical physiologically, with more muscularity in the dominant (right) arm than the non-dominant (left), the left upper arm has come to be used for MUAC measurement (21). The arm was then flexed 90 degrees from the elbow, and the tape was wrapped around the midpoint, not too tight or too loose. Two measurements were taken and the average was recorded as the final MUAC. The measurements were taken to the nearest 0.1 cm. Three BSc nurses as data collectors and two master-holder public health professionals as supervisors were recruited to collect the data.

### Study variables

Dependent variable: Undernutrition.

Independent variables: Sociodemographic variables (age, marital status, educational status, occupation, residence, and family member size), maternal reproductive and healthcare-related variables (age at first pregnancy, ANC follow-up, gravidity, birth interval, place of birth, frequency of breastfeeding, maternal illness, maternal meal frequency, and sources of food), and minimum dietary diversity score.

### Operational definitions

Undernutrition: MUAC of less than 23 cm (22).

Illness in the past 2 months: If a lactating, mother encountered any type of illness after entering the camps in the previous 2 months.

Minimum dietary diversity for women (MDD-W): Is defined using the ten food group categories according to the recommendation of the Food and Agriculture Organization (FAO) and the United States Agency of International Development (USAID) (23). The mother was asked what type of food she ate in the 24 h preceding the survey, independent of portion quantity. The Minimum Dietary Diversity Score (MDDS) is classified as;

Low: If the mother consumed <5, food groups out of the 10 food groups.

Adequate: If the mother consumed ≥5, food groups out of the ten food groups.

### Data processing and analysis

Data were checked for completeness, entered into EPI data (version 3.1), and exported to Statistical Package for Social Science (SPSS) (version 20) for statistical analysis. Descriptive statistics (mean and standard deviation for continuous variables; and frequencies and percentages for categorical variables) were computed. All variables were checked for normality and fulfillment of assumptions using histograms, boxplots, and scatter plots before analysis. In this study, the outcome variable was nutritional status coded 0 as undernutrition and 1 as normal (well nutrition). Binary logistic regression was used to identify the crude relationship of factors associated with undernutrition in lactating mothers, then variables significant at P < 0.25 were entered into the final multivariate model to identify significant factors independently predicting undernutrition among lactating mothers. Both crude odds ratio (COR) and adjusted odds ratios (AOR) together with their corresponding 95% confidence intervals were computed to assess the strength of association between the outcome and independent variables. In the multivariate analysis, variables with a p-value of less than 0.05 were considered significant. In the final model, the Hosmer–Lemeshow test was used to determine model fitness, and a value greater than 0.05 was considered a good fit.

### Data quality management

To assure data quality, all data collectors and supervisors received training and proper orientation before the actual commencement of data collection. To maintain consistency, the English version of the questionnaire was translated into the local language (Amharic) and then back into English. Two weeks before the actual data collection, the questionnaire was pre-tested on 5% of the study population at other sites with a similar population (Ebinat IDP site), and a few modifications were made. Furthermore, the accuracy, clarity, and completeness of data were reviewed on daily basis by the supervisors and principal investigators.

## Results

### Sociodemographic characteristics of respondents

A total of 420 lactating mothers participated in this study with a response rate of 99.5%. The mean (± SD) age of study participants was 26.5 (±5) years ranging from 17 to 45 years. Nearly half (51.4%) of the respondents were found in the age group of 25–34. The majority of the study participants (85.7%) were married, 38.6% of them had no formal education, and 52.8% were residing in urban areas. One hundred fifty-nine study participants had at least two under-five children in their households (Table 1).

**TABLE 1:** Sociodemographic characteristics of lactating mothers in Sekota Internally Displaced Persons (IDP) camps, northern Ethiopia, 2022.

Variables	Category	Frequency (n)	Percentage (%)
Age in a year	15–24	159	37.9
	25–34	216	51.4
	≥35	45	10.7
	Mean (± SD)	26.5 (±5)	
Marital status	Married	360	85.7
	Divorced	45	10.7
	Widowed	15	3.6
Educational status	No formal education	162	38.6
	Primary (1–8)	132	31.4
	Secondary (9–12)	69	16.4
	Above secondary (12^+^)	57	13.6
Occupational status	Housewife	228	54.3
	Government employee	54	12.9
	Private employee	96	22.9
	Others*	42	10
Family size	≤4	300	71.4
	5–8	108	25.7
	≥9	12	2.9
	Mean (± SD)	4.2 (± 1.6)	
Number of U-5 children in the household	One child	261	62.1
	Two or more	159	37.9
Sex of child	Male	273	65
	Female	147	35
Age of child (in a month)**	<6	87	20.7
	6–11	33	7.9
	12–23	99	23.6
	≥24	201	47.9
	Mean (± SD)	19.3 (±12.7)	

*Merchant, daily laborer; **Age of breast-feeding child recorded in a month.

### Maternal reproductive and healthcare-related characteristics

Less than half (48.6%) of lactating mothers attended antenatal care (ANC) follow-ups in their last pregnancy. The majority of the study participants (63.6%) had their first pregnancy while still in their teenage. One hundred eighty (42.9%) study participants had a birth interval of fewer than 36 months. More than half of the participants (52.9%) gave birth to their current child at home. The majority (84.3%) of study participants obtained their food from various groups that provide food assistance (Table 2).

**TABLE 2:** Maternal reproductive and healthcare-related characteristics among lactating mothers in Sekota Internally Displaced Persons (IDP) camps, northern Ethiopia, 2022.

Variables	Category	Frequency (n)	Percentage (%)
Age at first pregnancy	<20 years	267	63.6
	≥20 years	153	36.4
Antenatal care (ANC)	Yes	204	48.6
	No	216	51.4
Gravidity	Primi-gravida	144	34.3
	(2–4)	222	52.9
	≥5	54	12.9
Birth interval	First child	147	35
	<24 month	180	42.9
	≥24 month	93	22.1
Birthplace of current child	Home	222	52.9
	Institution	198	47.1
Frequency of breastfeeding/day	<8 times	318	75.7
	≥8 times	102	24.3
Illness in the past 2 months	Yes	105	25
	No	315	75
Maternal meal frequency/day	≤ two meals	72	17.1
	Three meals	258	61.4
	> three meals	90	21.4
Sources of food	Purchasing	36	8.6
	Food aid	354	84.3
	Others	30	7.1

### Dietary diversity characteristics of respondents

Regarding the food groups consumed by lactating mothers in the previous 24 h, all women consumed starchy staples. Whereas 80.5 and 76.9% of respondents consumed legumes and nuts, and other vegetables, respectively 24 h preceding the survey. More than one-third (42.4%) of lactating mothers’ minimal dietary diversity score was low or inadequate (Table 3).

**TABLE 3:** Dietary diversity frequency among lactating mothers in Sekota Internally Displaced Persons (IDP) camps, northern Ethiopia, 2022.

Food groups consumed in the previous 24 h		Number (percent)
Starchy staples		420 (100)
Legumes and nuts		338 (80.5)
Diary		165 (39.3)
Organ meat		147 (35)
Any egg		45 (10.7)
Meat, poultry, and fish		167 (39.8)
Dark green leafy vegetables		249 (59.3)
Other vitamin A-rich fruits and vegetables		264 (62.9)
Other vegetables		323 (76.9)
Other fruits		3 (0.7)
Minimum dietary diversity score (MDDS)	Low	178 (42.4)
	Adequate	242 (57.6)

### Nutritional status of lactating mothers

The nutritional status of lactating mothers was assessed using MUAC. Accordingly, 54.8% (95% CI: 49.9, 59.6) of lactating women were undernourished (Table 4).

**TABLE 4:** Nutritional status among lactating mothers in Sekota Internally Displaced Persons (IDP) camps, northern Ethiopia, 2022.

Variables	Category	Frequency	Percentage	95% CI
MUAC (cm)	<23	230	54.8	49.9, 59.6
	≥23	190	45.2	40.4. 50.1
	Mean (± SD)	22.6 ± 1.5		

### Factors associated with undernutrition among lactating mothers

The Hosmer–Lemeshow test was used to determine binary regression model fitness. The model fits with a P-value of 0.863 (Table 5). Then, to identify factors associated with undernutrition among lactating mothers in the study population, bivariate and multivariable logistic regression analyses were performed. MUAC was used to determine whether lactating women were undernourished (MUAC < 23 cm) or not (MUAC ≥ 23 cm). On binary logistic regression analysis, maternal age, family size, gravidity, birth interval, the birthplace of the current child, maternal illness in the past 2 months, maternal daily meal frequency, source of food, and minimum dietary diversity score were significantly associated with undernutrition at a p-value of < 0.25. However, in the final multivariate logistic regression model, age, family size, birth interval, daily maternal meal frequency, and dietary diversity score were significantly associated with undernutrition at a p-value of 0.05 (Table 6). Based on the principle of the logistic regression model, we used techniques like the normative category, the smallest value, and the category whose mean is in the middle to choose the reference category (24).

**TABLE 5:** Hosmer and Lemeshow goodness of fit test result.

Step	Chi-square	Df	Sig.
1	4.993	8	0.863

**TABLE 6:** Factors associated with undernutrition among lactating mothers in Sekota Internally Displaced Persons (IDP) camps, northern Ethiopia, 2022.

Variables	Category	Undernourished (MUAC < 23 cm)		COR (95% CI)	P-value	AOR (95% CI)	P-value
		**Yes**	**No**				
Age (in a year)	15–24	99	60	0.94 (0.44, 1.99)	0.703	0.21 (0.07, 0.65)	0.006*
	25–34	104	112	0.07 (0.02, 0.31)	0.012	0.20 (0.07, 0.57)	0.002*
	≥35	27	18	1		1	
Family size	≤4	182	118	1		1	
	(5–8)	45	63	0.05 (0.01, 0.36)	0.004	1.69 (0.30, 4.21)	0.240
	≥9	3	9	4.14 (1.74, 9.88)	0.001	4.35 (1.32, 10.22)	0.004*
Gravidity	Primi-gravida	96	48	0.32 (0.06, 1.65)	0.174	0.35 (0.05, 2.43)	0.370
	(2–4)	113	109	0.50 (0.06, 4.03)	0.514	1.56 (0.58, 4.12)	0.330
	≥5	21	33	1		1	
Birth interval	<24 month	83	97	4.60 (1.47, 9.93)	0.016	4.85 (1.24, 10.00)	0.012*
	≥24 month	45	48	1		1	
Place of birth	Home	119	103	0.35 (0.14. 0.84)	0.020	0.73 (0.45, 1.17)	0.388
	Institution	111	87	1		1	
Illness in the past 2 months	Yes	66	39	1.48 (0.77, 2.84)	0.244	1.35 (0.76, 2.40)	0.430
	No	164	151	1		1	
Maternal meal frequency/day	≤ two meals	45	27	2.98 (1.00, 8.84)	0.049	2.54 (1.12, 5.75)	0.016*
	Three meals	152	106	1.10 (0.48, 2.52)	0.818	0.98 (0.67, 3.94)	0.056
	> three meals	33	57	1		1	
Sources of food	Purchasing	9	27	0.23 (0.04, 1.39)	0.109	0.12 (0.03, 0.40)	0.487
	Food aid	200	154	0.08 (0.01, 0.47)	0.006	0.48 (0.18, 1.28)	0.070
	Others	21	9	1		1	
MDDS	Low	106	72	1.78 (0.81, 3.99)	0.153	1.79 (1.03, 3.10)	0.019*
	Adequate	124	118	1		1	

*Significant at a *p*-value of < 0.05 in multivariate logistic regression analysis, 1 indicates reference category, MDDS is minimum dietary diversity score.

## Discussion

The prevalence of undernutrition among lactating mothers was found to be 54.8% in this study. In comparison to the findings of this study, most Ethiopian studies reported a lower prevalence of undernutrition among lactating women, such as in the Afar region 33.3% (12), Angecha district 21.2% (13), Arba Minch districts 17.4% (14), Dega Damot district 21.8% (15), Dessie town 21% (16), Moyale district 17.7% (25), and Shebedino district, Sidama region 25.9% (26). The possible reason might be due to the different sociodemographic and economic natures of the study population. It could also be because our study participants are displaced individuals as a result of the civil war, which has had a significant impact on their agricultural productivity and economy in comparison to other regions of the country that have been stable without civil conflicts (27). The finding is even greater than that of a study conducted in the Bale zone of Oromia regional state, which revealed 24% undernutrition among lactating women in a humanitarian setting (17). The higher prevalence in the current study could be attributed to insufficient humanitarian support by the national government or international humanitarian groups in the selected IDP camps.

Predictors of undernutrition among lactating mothers are maternal age, family size, birth interval, maternal meal frequency, and MDDS (Table 6). In this study, young age (<35 years old) has a significantly low risk of undernutrition than those older ages. This could be due to biological changes caused by aging, such as changes in body composition and energy calories contributing to an increased risk of undernutrition (28). Lactating mothers with a family size of ≥9 are 4.35 times more likely to be malnourished than those with smaller family sizes. The finding is similar to the studies conducted in Nekemte (Oromia region) and Wenberma district (Amhara region), Ethiopia (29, 30). The possible reason could be due to the household food insecurity issue in women with large family sizes resulting in undernutrition (31). In this study, lactating mothers with short birth intervals (<24 months) were 4.85 times more likely to be undernourished than their counterparts. This is consistent with the studies conducted in the Afar region (12) and Arba Minch district (14). It might be due to the recurrent loss of macronutrients and micronutrients from the woman’s body during pregnancy, delivery, and breastfeeding as a result of short birth spacing and repeated childbearing. Additionally, a short birth interval does not provide the mother with enough time to recover from the nutritional burden (32). Short birth intervals have also been associated with adverse outcomes in a woman’s nutritional status, such as nutritional depletion, folate depletion, and micronutrient insufficiency (33). Lactating mothers with a low daily meal frequency (≤2 meals) were 2.54 times more likely to be malnourished than those who consumed more frequent feeding. MDDS was also statistically associated with undernutrition among lactating mothers. Lactating mothers with inadequate MDDS were approximately twice more malnourished as those with adequate MDDS. The finding is consistent with the study done in the Afar region (12), Dessie town (16), and Angecha district (13). Taking at least two extra meals per day during lactation is recommended by the essential nutrition action (ENA) for all lactating women (34). Poor dietary intake is clearly one of the immediate causes of malnutrition, even in lactating women. It was also demonstrated that inadequate minimal dietary diversity among lactating mothers is a potential cause of maternal undernutrition since consumption of a variety of food types gives various vital nutrients for optimum growth (35). As a result, dietary sufficiency and a varied diet are crucial for lactating mothers throughout the postpartum period (36). Furthermore, dietary deficiency combined with a lack of diversity may jeopardize maternal health, worsening malnutrition in lactating mothers (37).

Our study, however, has the following limitations. First, we did not include the wealth index, household food security, and the presence of comorbidities (health status) of participants as independent factors, which might have caused a confounding effect. Second, dietary diversity and meal frequency were examined using a 24-h recall, which may not represent the study participants’ regular intake.

## Conclusion

The prevalence of undernutrition among internally displaced lactating mothers in Sekota IDP camps was found to be very high. Older Age, large family size, short birth interval, maternal meal frequency, and low dietary diversity score are the factors associated with undernutrition. Governments and other concerned organizations involved in providing care and support to Sekota IDP camps should increase their efforts to improve the nutritional status of lactating mothers.

## Data availability statement

The original contributions presented in this study are included in the article/supplementary material, further inquiries can be directed to the corresponding author.

## Ethics statement

The studies involving human participants were reviewed and approved by from the Debre Tabor University College of Health Sciences Research Ethics Review Committee. The patients/participants provided their written informed consent to participate in this study.

## Author contributions

MM and MTA conceived and designed the study. MW and EC were actively involved in the guidance of the conception of research and critical review of the manuscript. YB, TA, and MA were involved in the proposal development and data collection. MM was actively working on data analysis, and writing the research report, and was a major contributor to drafting the manuscript. All authors have read, provide critical feedback, and approved the final manuscript.
